# What hypertensive patients want to know [and from whom] about their disease: a two-year longitudinal study

**DOI:** 10.1186/s12889-020-8421-6

**Published:** 2020-03-12

**Authors:** Erika R. Cappelletti, Andrea Greco, Alessandro Maloberti, Cristina Giannattasio, Patrizia Steca, Marco D’Addario

**Affiliations:** 1 Health Promotion Division, ATS Milan, Via Juvara, 22, 20129 Milan, Italy; 2 Department of Human and Social Sciences, University of Bergamo, Bergamo, Italy; 3 Cardiology 4, “A. De Gasperis” Department, ASST GOM Niguarda Ca’ Granda Hospital, Milan, Italy; 4 School of Medicine and Surgery, University of Milan-Bicocca, Milan, Italy; 5 Department of Psychology, University of Milan-Bicocca, Milan, Italy

**Keywords:** Information needs, Hypertension, Health information sources, Longitudinal research

## Abstract

**Background:**

This study explored both the evolution of the information needs and the perceived relevance of different health information sources in patients with essential hypertension. It also investigated the relationships between information needs and the perceived relevance of information sources with socio-demographic and clinical variables.

**Methods:**

Two hundred and two patients with essential arterial hypertension were enrolled in the study and evaluated at baseline and during three follow-ups at 6, 12 and 24 months after baseline. Patients had a mean age of 54.3 years [range 21–78; SD = 10.4], and 43% were women. Repeated measures ANOVA, Bonferroni post hoc tests, and Cochran’s Q Test were performed to test differences in variables of interest over time.

**Results:**

It was observed a significant reduction in all the domains of information needs related to disease management except for pharmacological treatment and risks and complications. At baseline, patients reported receiving health information primarily from specialists, general practitioners, relatives, and television, but the use of these sources decreased over time, even if the decrease was significant only for relatives. Multiple patterns of relationships were found between information needs and the perceived relevance of sources of information and socio*-*demographics and clinical variables, both at baseline and over time.

**Conclusions:**

The findings showed a general decrease in both the desire for information and the perceived relevance of different information sources. Hypertensive patients appeared to show little interest in health communication topics as their disease progressed. Understanding patients’ information needs and the perceived relevance of different information sources is the first step in implementing tailored communication strategies that can promote patients’ self-management skills and optimal clinical outcomes.

## Background

Hypertension is globally the strongest modifiable risk factor for cardiovascular disease (CVD) and related disability; it causes 9.4 million deaths worldwide every year [1] and it remains the leading risk factor for disability-adjusted life years (DALYs) [2]. Despite extensive knowledge about ways to both prevent and treat hypertension through individual lifestyle changes, healthy behaviors and medication adherence are still suboptimal, leading to adverse cardiovascular effects [3–6].

To improve management of hypertension, the Lancet Commission issued a 10-point action plan in which *a key* one was to improve communication between provider and patient and to tailor education about hypertension throughout the life course [7]. Effective health communication is fundamental to achieving optimal adherence to recommended health behaviors and treatment [8, 9]. Extensive research has shown that as the information provided becomes more tailored to the personal features of the patients, it becomes more effective in influencing their behaviors [10–12]. In the design and delivery of tailored health messages, two key variables are patients’ information needs and preferences for sources of information, but the role of these indicators have not been sufficiently investigated [13, 14]. Especially in the case of chronic diseases, meeting patients’ information needs and preferences is positively associated with their global satisfaction, quality of life, psychological well-being, and improved health status [15, 16]. Health providers perceive information needs differently than patients. When patients’ needs are left unresolved, lower adherence rates may result [17].

Research in this field found that patients with acute coronary syndrome, myocardial infarction, and heart failure judge all types of information as important, with a preference for information on medication (names, dosage and side effects), risk factors (especially how to modify incorrect behaviors), and physiology (knowing how to manage signs and symptoms) [18–21]. However, the majority of researches has been conducted using a cross-sectional methodology; few studies have been conducted using a longitudinal approach and there is still a lack of knowledge about how the specific need for health information changes as the disease progresses. One recent study that evaluated change in information needs over twenty-four months after the first diagnosis of acute coronary syndrome showed a reduction in information needs, but this decrease was significant only for topics related to daily life activities, behavioral habits, and risk and complications [22]. These results suggest that information needs do not represent stable interests; rather, they change across the different moments of the disease.

In addition to the content of health messages, a crucial role is played by the sources through which the information is delivered. Today, information on how to correctly manage hypertension is available from multiple sources, such as expert opinions, web pages, media, blogs, personal experience, and books/ journals/magazines. This plurality of sources implies the need for updated knowledge on patients’ use and trust in various sources of information to better deliver health information. Nevertheless, a few studies have been conducted, with limitations in terms of sample size and heterogeneity of composition [not only patients but also non-clinical population, like medical students]. Moreover, these studies have found inconsistent results: some have shown that traditional mass media such as television, radio, and newspapers were major information sources [23, 24], whereas others have reported that people’s primary hypertension information sources were their doctors and relatives [25, 26].

Understanding patients’ information needs and preferences for sources of information is crucial to help health care providers in giving the right information at the right time in order to tailor health messages and, thus, make communication relevant for the patients. To the best of our knowledge, no studies have been conducted on patients affected by hypertension, especially through the application of a longitudinal approach. Hence, the purpose of the study reported here was to investigate levels of and change over time in hypertensive patients’ self-reported need for information about the disease and the perceived relevance of different sources of information. A further aim was to explore the relationships between need and preferences with socio-demographic and clinical variables.

Due to the exploratory nature of the study and the scarcity of previous studies on the issue addressed, it was difficult to develop specific research hypotheses. Based on previous studies with different populations (e.g. [22]), it was hypothesized that the need for information would change over a two-year period, with a greater need for information on risk and complications and drug treatment at baseline and an increased desire for information on disease management as time progresses. It was also hypothesized that the primary source of information would be health care practitioners. No hypotheses about the role of socio-demographic and clinical variables were developed.

## Methods

This is a secondary analysis from a multisite, longitudinal study of personality, resilience and self-regulation process on a large cohort of ACS and hypertensive patients in Italy. The research methodology was the same used in previous studies [22, 27, 28].

### Participants and procedure

Patients who were already receiving pharmacological treatment or had a diagnosis of essential arterial hypertension (SBP > =140 mmHg and/or DBP > =90 mmHg) were recruited between January 2011 and April 2012 during their regular cardiological examinations in the Clinica Medica (medical clinic) unit of a hospital in Northern Italy. Patients were selected by convenience sampling method and they were eligible for this study if they met the following inclusion criteria: > 30 years old; good understanding of the Italian language; no moderate-severe cognitive impairment, psychiatric disorders or diseases with limited expected survival. Eligible patients were told about the aim of the study and its longitudinal design with three follow-ups at 6 (t1), 12 (t2), and 24 months after baseline (t3). After the sign of the informed consent form, a physician collected clinical data related to a) body mass index (BMI); b) waist circumference; c) blood pressure values; d) diabetes mellitus; e) the presence of different CVD risk factors, including gender, age, smoking behavior, dyslipidemia (abnormal level of total, high-density, and low-density lipoprotein cholesterol, and triglycerides), obesity, abdominal obesity, and family history of premature CVD. After the clinical examination patients answered some questions related to their need for health information and the perceived relevance of information sources. This procedure was repeated in the three follow-ups, during which a physician collected further clinical information related to the number of a) specialist visits, b) emergency room visits, c) hospitalizations related to hypertension, and patients’ blood pressure values.

The Ethical Committee of the University of Milan-Bicocca and of the healthcare center from which patients were recruited approved the study.

### Measures

#### Information needs

As done in a previous study with patients affected by acute coronary syndrome [22], information needs were evaluated with two questions that examined the need for additional information in one of six domains related to hypertension and its management: "Pharmacological Treatment"; "Knowledge About the Disease"; "Daily Activities"; "Behavioral Habits"; "Impact of the Disease"; "Risk and Complications". The first question asked patients to determine, on a five-point Likert scale ranging from 1 ("*I want to know nothing about the topic*") to 5 ("*I want to know everything about it*") the amount of additional information needed by the patients in the six domains ("*Indicate how much information you would like to receive about the following topics connected to the management of your cardiovascular disorders*"). The second question asked patients to judge the importance of the six domains assigning a score from 1 to 6 ("*Now please rate the importance of the topics listed below; you must assign a value from one for the most important topic to six for the least important one*). To avoid the propensity of patients to evaluate all knowledge as "very" or "extremely" important, a balanced index was calculated by multiplying the score on question 1 by the reversed score on question 2. The balanced index had a score range from 1 to 30 with higher scores indicating a greater need for information.

#### Information sources

Regarding sources of information, one dichotomous question investigated whether patients had received information from one of nine sources of information: “General Practitioners” (GPs), “Specialists”, “Relatives”, “Friends”, “Information Leaflets given by Physician”, “Information Leaflets given by Associations”, “Magazines”, “Internet”, and “Television”. A second question asked patients to assess, on a five-point Likert scale ranging from 1 (“not at all”) to 5 (“very relevant”), the perceived relevance of the nine sources (“*Think about how you have learned about your disease from the time you became aware you had the illness. For each of the sources listed below, indicate how relevant the source was in providing you with information*”).

#### Socio-demographic variables

Personal details were obtained about gender, age, marital and employment status, and education level.

#### Time from the diagnosis of hypertension

Patients were given an open-ended question on how many months/years it had been since they were diagnosed with hypertension *(“How long you been diagnosed with hypertension from a healthcare provider?”).* The responses that were reported in years were converted in months, and this variable was called “time from the diagnosis of hypertension”.

#### Total cardiovascular risk index” (TCRi)

For each patient a “Total Cardiovascular Risk Index” (TCRi) was determined based on the sum of the clinical data evaluated during clinical examination, with 1 point assigned for each cardiovascular risk factor present. Following the “2018 ESC/ESH Guidelines for the Management of Arterial Hypertension” [29] were considered risk factors: male sex, age [men > = 55 years; women > = 65 years], smoking, obesity [BMI > = 30 kg/m2 [height2]], abdominal obesity [waist circumference: men > = 102 cm, women > = 88 cm], diabetes mellitus, dyslipidemia [total cholesterol > 190 mg/dL and/or LDL-C > 115 mg/dL and/or HDL-C: men < 40 mg/dL, women < 46 mg/dL and/or triglycerides > 150 mg/dL], elevated blood pressure values [SBP > = 140 mmHg and/or DBP > = 90 mmHg], and a family history of premature CVD [men aged < 55 years; women aged < 65 years].

### Statistical analysis

Analyses of Variance (ANOVA) for repeated measures were performed to assess statistical differences among information needs and the perceived relevance of sources over time, with a check for sphericity using Mauchly’s test of sphericity. Post hoc tests (0.05) were conducted using Bonferroni analysis. Cochran’s Q-test was used to assess changes in the proportion of patients receiving information from information sources over the four time points. The relationships among socio-demographic (i.e., gender, age, marital and employment status, educational level) and clinical (i.e., time from the diagnosis of hypertension, SBP, DBP, and TCRi) variables, information needs and the perceived relevance of sources were analyzed using regressions analyses.

Missing data were substituted using hot deck imputation [30], a statistical procedure that replaces a missing value with the value of a similar “donor” in the dataset. This method is recommended when the percentage of missing data is lower than 10% regardless of the pattern of the missing data [31]. In this study the percentage of missing data was 0.3%. Therefore, values were imputed using hot deck imputation; only one case was excluded from the analysis. The “donor” was selected according to the gender and age of the participants.

The significant level was set at *p* ≤ 0.05 for all the analyses. Statistical Package for Social Sciences version 24.0 for Windows (SPSS Inc., Chicago, USA) was used to analyze the data.

## Results

### Participants’ characteristics

Two hundred and seventy-one consecutive patients were enrolled at baseline; twenty-five patients declined to participate at t1 (attrition rate = 9.2%), seventeen at t2 (attrition rate = 6.9%) and twenty-three at t3 (attrition rate = 10%). One patient died of causes not directly related to hypertension before t2, and three patients died before t3. To exclude any possible differences, the distributions of the collected variables for the 271 participants measured at baseline were compared between the sample used in the analysis (*N* = 202) and the dropouts (*N* = 69). The Mann-Whitney non-parametric test was used both because of the difference in the sample sizes of patients used in the analysis and the dropouts [32]. Patients who refused to participate at t1, t2 and t3 did not differ from the final study group with respect to socio-demographic variables, clinical data, information needs, and the perceived relevance of information sources, as evaluated at baseline.

Two hundred and two patients participated in this study. Patients had a mean age of 54.3 years (range 21–78; SD = 10.4), were mainly married (78.7%) with a high school degree (49%) and employed (56.4%); women were 42.6% of the sample. Table 1 shows full information about the demographic characteristics of the participants. The “*time from the diagnosis of hypertension*” variable varied from less than two months to more than thirty years.

**Table 1:** Patients’ sociodemographic characteristics

Characteristic	Value
Number of patients	202
Age, mean ± SD	54.3 ± 10.4
**Gender**	N (%)
Female	86 (42.6)
Male	116 (57.4)
**Education level**	N (%)
< High School Diploma	54 (26.7)
High School Diploma	99 (49)
> High School Diploma	49 (24.3)
**Employment Status**	N (%)
Employed	114 (56.4)
Retired	55 (27.2)
Unemployed	10 (4.9)
Housewife	12 (5.9)
Retired with some work activities	11 (5.4)
**Marital status**	N (%)
Married	159 (78.7)
Not Married (Also widowed/divorced)	43 (21.3)

Roughly half of the sample (45.8%) had family histories of CVD. Furthermore, slightly less than one-third, 30.2%, presented with obesity, 15.1% had dyslipidemia, and 8.1% presented with diabetes; moreover, 4.5% had had prior cardiac events, and 1.6% had nephropathy (Table 2).

**Table 2:** Patients’ clinical information

Clinical characteristic	mean ± standard deviation (SD)
Body Mass Index kg/m	26.7 ± 3.9
Waist Circumference cm	92.6 ± 12.2
Total Cholesterol mg/dl	200 ± 34.7
Glucose mg/dl	94 ± 19.8
Risk Factors	3 ± 1.5
**Risk Factors**	N(%)
Dyslipidemia	30 (15.1)
Smoking History	64 (32.2)
Diabetes	16 (8.1)
Obesity	61 (33.7)
Family History of premature CVD	88 (45.8)
Nephropathy	3 (1.6)
Prior cardiac events	9 (4.5)
Menopause	38 (19.9)
**Pharmacological Treatment**	N(%)
**Yes**	176 (87.1)

Table 3 reports patients’ blood pressure values, as recorded in all measurements, and the frequency of each rank, according to the blood pressure classification [29]. At baseline, half of the sample’s pressure values ranged between “Optimal” and “Pre-Hypertension” (56%); these values changed in the subsequent follow-ups. Table 3 also describes the number and percentage of patients who monitored their blood pressure at home during the three follow-ups of the study. As it can be seen, almost the totality of patients monitored their pressure at home.

**Table 3:** Blood Pressure Value

	Baseline	6 months follow-up (T1)	12 months follow-up (T2)	24 months follow-up (T3)
**Blood Pressure Value** Measured by Physician	Mean ± SD	Mean ± SD	Mean ± SD	Mean ± SD
SBP^a^, mean ± SD	134 ± 16.5	132 ± 16.2	131 ± 17.4	135 ± 17.4
DBP^b^, mean ± SD	82 ± 9.9	82 ± 10	81 ± 9.5	83 ± 10.3
**Blood Pressure Classification**	N (%)	N (%)	N (%)	N (%)
Optimal	17 (8.4)	38 (18.8)	41 (20.3)	32 (15.8)
Normal	48 (23.8)	42 (20.8)	46 (22.8)	36 (17.8)
Pre-Hypertension	48 (23.8)	47 (23.3)	53 (26.2)	52 (25.7)
Hypertension	89 (44)	75 (37.1)	62 (30.7)	82 (40.5)
Home Blood Pressure Monitoring	N (%) -	N (%) 196 (97)	N (%) 197 (97.5)	N (%) 198 (98)

^a^Systolic Blood Pressure

^b^Diastolic Blood Pressure

### Information needs

All the information needs showed a violation of the assumption of sphericity: “Pharmacological Treatment” (x^2^(5) = 14.73, *p* < .05), “Knowledge About the Disease” (x^2^(5) = 23.49, *p* < .001), “Daily Activities” (x^2^(5) = 29.13, *p* < .001), “Behavioral Habits” (x^2^(5) = 12.90, *p* < .05), “Impact of the Disease” (x^2^(5) = 21.76, *p* < .01), and “Risk and Complications” (x^2^(5) = 21.38, *p* < .01). The degrees of freedom were therefore adjusted using Greenhouse-Geisser estimates of sphericity (ε = .95, ε = .94, ε = .91, ε = .96, ε = .94, and ε = .94, respectively). Results showed that information need decreased over time for “Knowledge About the Disease”, “Daily Activities”, “Behavioral Habits”, “Impact of the Disease”, while no reduction was found for “Pharmacological Treatments”, and “Risk and Complications”.

Table 4 presents the mean scores, standard deviation, test F and *p* levels.

**Table 4:** Information needs over time

Information Need	Mean (SD) at Baseline	Mean (SD) at 6 months follow-up (T1)	Mean (SD) at 12 months follow-up (T2)	Mean (SD) at 24 months follow-up (T3)	df	F
**Pharmacological treatment**	15.6 (8.6)	14.2 (8.3)	14.3 (8.4)	15.2 (8.4)	2.8;573.6	2.11
**Knowledge About the Disease**	16.9 (8.5)	15.4 (8.4)	15.1 (8.4)	14.3 (8.3)	2.8;565.1	6.59***
**Daily Activities**	12.6 (7.5)	11.4 (7.5)	10.4 (7.1)	10.5 (6.7)	2.7; 550.8	5.61**
**Behavioral Habits**	13.0 (7.3)	12.5 (7.4)	11.1 (6.7)	11 (6.9)	2.9;577.8	5.52**
**Impact of the Disease**	12.7 (7.5)	11.2 (7.1)	11.2 (7.2)	10.7 (7)	2.8;567.2	3.89**
**Risk and Complications**	15.6 (7.9)	15.5 (7.8)	15.4 (8.3)	15.1 (8)	2.8;562.5	.26

Note: ** Significant differences (p < .01); *** Significant differences (p < .001)

**Table 5:** Number and percentage of patients that have received information from a source over time

Information Sources	Baseline	6 months follow-up (T1)	12 months follow-up (T2)	24 months follow-up (T3)	df	Cochran’s Q
**General Practitioners**	174 (86.1)	141 (69.8)	115 (56.9)	132 (65.3)	3	60.16***
**Specialists**	187 (92.6)	155 (76.7)	122 (60.4)	149 (73.8)	3	69.87***
**Relatives**	159 (78.7)	140 (69.3)	117 (57.9)	129 (63.9)	3	35.25***
**Friends**	106 (52.5)	94 (46.5)	62 (30.7)	86 (42.6)	3	33.06***
**Information Leaflets-Physician**	108 (53.5)	100 (49.5)	70 (34.7)	89 (44.1)	3	22.95***
**Information Leaflets Associations**	60 (29.7)	60 (29.7)	42 (20.8)	67 (33.2)	3	12.43**
**Magazines**	115 (56.9)	112 (55.4)	100 (49.5)	112 (55.4)	3	4.18
**Internet**	113 (55.9)	112 (55.4)	102 (50.5)	99 (49)	3	5.29
**Television**	128 (63.3)	115 (56.9)	109 (54)	111 (55)	3	7.93*

Note: *Significant differences (p < .05);** Significant differences (p < .01); *** Significant differences (p < .001)

### Information sources

At baseline patients received information from “Specialists” (92.6%), “GPs” (86.1%), “Relatives” (78.7%), and “Television” (63.3%). Roughly half of the sample received information from “Magazines” (56.9%), “Internet” (55.9%), “Information Leaflets given by Physician” (53.5%), and “Friends” (52.5%). Only less than one third of the sample received information from “Information Leaflets given by Associations” (29.7%). Table 5 presents the number and percentage of patients that have received information from a source over time.

Results showed a reduction in information provision for almost all the sources during the three follow-ups. The Cochran’s Q test indicated that this reduction was significant for: “GPs” (x^2^(3) = 60.16, *p* < .001); “Specialists” (x^2^(3) = 69.87, *p* < .001); “Relatives” (x^2^(3) = 35.25, *p* < .001); “Friends” (x^2^(3) = 33.06, *p* < .001); “Information Leaflets given by Physician” (x^2^(3) = 22.95, *p* < .001); “Information Leaflets given by Associations” (x^2^(3) = 12.43, *p* < .01); “Television” (x^2^(3) = 7.93, *p* < .05). Only for “Magazines” (x^2^(3) = 4.18, *p* = .243) and “Internet” (x^2^(3) = 5.29, *p* = .152) the reduction between the different time points was not significant.

Mauchly’s test showed a violation of the assumption of sphericity for: “GPs” (x^2^(5) = 16.85, *p* < .01), “Specialists” (x^2^(5) = 15.08, *p* < .01), “Information Leaflets given by Physician” (x^2^(5) = 11.52, *p* < .05), “Television” (x^2^(5) = 14.65, *p* < .01)). The degrees of freedom were therefore adjusted using Greenhouse-Geisser estimates of sphericity (ε = 88, ε = 91, ε = 80, and ε = 89, respectively).

**Fig. 1:**
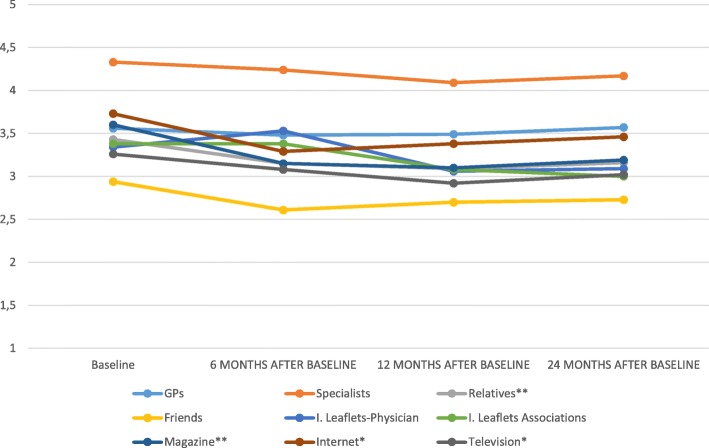
Changes in perceived relevance of health information sources over time. Note: *Significant differences (*p* < .05);** Significant differences (*p* < .01)

Through a repeated measures ANOVA it was found a significant decrease in the perceived relevance for “Relatives” (F(3;240) = 4.28, *p* < .01); “Magazines” (F(3;153) = 5.99, p < .01), “Internet” (F(3;153) = 3.61, *p* < .05), and “Television” (F(2.66;159.63) = 3.10, *p* < .05); no significant changes were found for the other sources (Fig. 1).

### Relationship between information needs, information sources, socio-demographics, and clinical variables

At baseline, more educated patients desired less information about “Knowledge About the Disease” (*β* = −.183, *p* < .05) and more information about “Behavioral Habits” (*β* = .164, *p* < .05) and “Daily Activities” (*β* = .176, *p* < .05). Employment status was related to the information need about “Knowledge About the Disease” and “Daily Activities”; in particular, housewives wanted more information on the first topic (*β* = −.155, *p* < .05), while retired patients and retired patients who had some work activities desired more information on daily life activities (*β* = .231, *p* < .05; *β* = .230, *p* < .05, respectively); retired patients who had some work activities also desired more information on “Behavioral Habits” (*β* = .247, *p* < .01). Age and SBP were related to “Impact of the Disease” (*β* = −.305, *p* < .01 and *β* = .228, *p* < .05, respectively), with patients who were younger or had higher levels of SBP who were more interested in information on how to handle hypertension-related stress. Gender was related to “Knowledge About the Disease” (*β* = .180, *p* < .05): women desired more information on the anatomical/functional nature connected to hypertension. No relationships were found between time from the diagnosis of hypertension, DBP, TCRi and information need.

Repeated measure ANOVA showed that the information need about “Knowledge About the Disease” was related to patients’ marital status (F(2.80;467.85) = 3.36, *p* < .05), with married patients who wanted more information over time. Gender was related to the information need about “Daily Activities” (F(2.73;455.55) = 3.30, *p* < .05), with women who wanted more information over time. *“*Impact of the Disease” was related with SBP (F(2.79;465.54) = 2.89, *p* < .05), with patients with higher levels of SBP who were more interested in information on how to handle hypertension-related stress. “Risk and Complications” was related to DBP (F(2.82;468.28) = 3.20, *p* < .05), with patients with higher levels of DBP who were more interested in information on this topic. No relationships were found with employment status, educational level, time from the diagnosis of hypertension, and TCRi.

The perceived relevance of “GPs” was related to education (*β* = −.205, *p* < .05) and employment status (*β* = −.255, *p* < .05), with more educated and retired patients who perceived this figure as less relevant. Age was related to the perceived relevance of “Specialists” (*β* = .246, *p* < .05), with older patients who perceived this source as more relevant. Education level and employment status were related to “Family”, (*β* = .270, *p* < .05) with more educated and retired patients who perceived this source as more relevant. Age, employment status and time from the diagnosis of hypertension were positively related to the perception of relevance of “Information Leaflets given by Associations” (*β* = −.441, *p* < .05; *β* = .536, *p* < .01; *β* = .317, *p* < .05, respectively), with younger patients, retired patients and who had a longer history of the disease who perceived information from this source as more relevant. Gender was related to the perception of relevance of “Magazines” (*β* = .360, *p* < .01), with women who perceived this source as more relevant. Older patients perceived as less relevant information from “Internet” (*β* = −.293, *p* < .05). No relationships were found between SBP, DBP, TCRi and the perceived relevance of information sources.

When the relationships between information sources and demographic and clinical variables were analyzed over time, employment status was associated with the perceived relevance of “Relatives” (F (12; 174) = 1.92, *p* < .05). DBP was positively associated with the perceived relevance of “Internet” (F (3; 102) = 3.18, p < .05). Time from the diagnosis of hypertension was positively associated with the perceived relevance of “Relatives” (F (3; 174) = 2.75, *p* < .05). No relationships were found with gender, age, education, marital status, SBP, and TCRi.

## Discussion

The current study aimed to investigate hypertensive patients’ need for information about their disease and the perceived relevance of different sources of information and how these variables change over twenty-four months. The results showed a general decrease in both desired information and the perceived relevance of sources over time. Patients desired less information on their pathology and on how to self-manage it, whereas they continued to desire medical information related to medical treatment and complications of the disease. This preference for medical information compared with lifestyle information has been identified in previous research [21, 22, 33–36] and deserves attention by health practitioners. Research continuously shows that patients fail to adhere correctly to medical advice or to change their unhealthy behaviors [37–39]; thus, it is essential to help them understand what they can do to self-manage their health condition and to prevent complications. The fact that patients report being less interested in information about behavioral habits and daily life activities is discouraging and should prompt research to find new ways to communicate this important knowledge.

The higher interest in the risks and complications of hypertension could indicate patients’ fears and worries about the possible worsening of their health condition; however, the participants reported no interest in what they could do in terms of self-care behaviors to reduce these complications. These results likely suggest that patients did not fully understand the degree to which lifestyle changes are necessary to manage hypertension and their general condition. It could be supposed that patients’ need for information on relevant topics decreases over time because they have already a full understanding of their disease. However, previous research showed that hypertensive patients have a knowledge deficit and held erroneous explanations for their hypertension [40]. Future studies could consider the role exercised by the amount of information already known by patients in the self-reported need for information about the disease.

Regarding information sources, the results showed that at baseline patients’ major sources are health care providers, both GPs and specialists, followed by relatives and television. Roughly half of the sample reported having received information from magazines, internet and information leaflets given by physician. Only brochures given by associations were irrelevant in informing patients, probably because this kind of material is not widely widespread. The lower use of magazines, internet, and information leaflets compared with interpersonal sources of information could be indicative of patients’ general tendency to not actively search for information. In fact, these sources give information to people who search for it. Besides, interpersonal sources are more reassuring and allow patients to take some control over their health. This result is consistent with previous studies [22, 23, 41]. Surprisingly, the television was reported to be more informative compared to the Internet. This result is inconsistent with previous studies [25], in which the Internet was a widely used source of information for the management of long-term conditions, but it is similar to results found by Stavropoulou [23] with Greek patients affected by hypertension. Yet, it is not a surprising finding given that Italy lags behind other European countries in the use of the Internet [42]. Even the sample’s age [mean = 54; higher than in Akter et al., 2014] and education [25% of the sample had not obtained a high school diploma; lower than in Akter et al., 2014] could explain this result. This hypothesis is also confirmed by the significant relationship found between age and the perceived relevance of the Internet, with older patients who perceived as less relevant information from this source. Regarding patients’ perception of relevance for the multiple sources, results showed a significant decrease in the relevance for relatives, magazines, internet, and television. It is important to note that the scores for the majority of the sources are above level three, indicating that these sources are perceived to be fairly significant.

Multiple patterns of relationships emerged between socio-demographic characteristics and variables under analysis. Higher educational level was significantly associated with needing more information on knowledge of the disease and behavioral habits. This could reveal a greater understanding of their crucial role in disease management in patients with higher education. Age and SBP were related to the need for information on how to manage the distress related to hypertension, with patients who were younger or had higher levels of SBP who were more interested in information on how to control distress related to the disease. The marital status was related to a greater need for information on knowledge about the disease over time. Despite researches have shown that relatives often play a crucial role in patients’ hypertension self-management [43–46] they are rarely included in the patient-physician discussions. Information appeared to be the greatest need of family members of critically ill patients [45]. It is possible that the greater information need in married patients was influenced by the need for information of the spouse. This hypothesis should be investigated in future researches. Gender was related with the need for information on daily life activities over time, with women who desire more information on this topic. This result is consistent with a previous study [47]. The level of DBP was associated with the need for information about possible risk and complications due to the disease. This could reveal a higher level of worries in patients with mayor severity of hypertension. This hypothesis should be investigated in future research.

Regarding information sources, some relationship arose at baseline with information sources and the socio-demographic and clinical variables related to gender, age, education level, and time from the diagnosis of hypertension. However, these relationships disappeared when analyzed over time, except for the time from the diagnosis of hypertension, that indicated patients’ history of the disease, that was positively associated with the perceived relevance of family. It was thought that this variable was more related to patients’ need and the perceived relevance of sources. Future researches should deepen the possible relationships between health communication topics and clinical variables.

Health information needs and sources of information are under-investigated areas. To the best of our knowledge, this is one of the first studies to provide information on the current state of patients’ information needs and preferences for sources of information with respect to hypertension and on how these variables change as the disease progresses. The two-year longitudinal design and the large sample size represent the study’s strengths. Despite its merits, it also presents some limitations. First, the generalizability of these findings could be limited because patients were recruited from a single health care center where they were being followed to manage their disease. Furthermore, only patients’ socio-demographic and clinical variables were considered as possible factors correlated to needs and preferences; other variables associated with needs for health information and health outcomes such as health literacy [48, 49] and psychological variables [28] were not assessed in this study. Additionally, the use of volunteer participants may likely have resulted in an overrepresentation of those who were more interested in the topics analyzed. Future research should consider population-based surveys to limit the effect of this possible bias.

## Conclusions

The results here presented have multiple implications for health professionals involved in developing interventions to improve patients’ adherence and behaviors. Educational, communicational, and awareness-raising interventions should provide patients with information, education or skills to modify their unhealthy behaviors. Education and communication are related tools that offer great potential to improve the global management of elevated blood pressure, helping the patients to understand their condition and their role in the healthcare process.

Considering patients’ needs, preferences, and the change in these variables over time will allow professionals to deliver the correct information at the right moment, avoiding misconceptions and misinformation. The study shows that there are areas of information, such as behavioral habits, that patients consistently place as a low priority yet are crucial to patients’ overall well-being. New and better ways to deliver information should be taken into account, and patients need to be educated about the importance of the information received to enable them to focus on primary and secondary prevention.

It is highly recommended that research on patients’ information needs and preferences continues to be conducted, especially for those diseases that have been under-investigated, such as hypertension.

## Data Availability

Not applicable.

## Abbreviations

ACS: Acute Coronary Syndrome
ANOVA: Analysis of Variance
BMI: Body Mass Index
CKD: Chronic Kidney Disease
CVD: Cardiovascular Disease
DALYS: Disability-Adjusted Life Years
DBP: Diastolic Blood Pressure
GP: General Practitioner
SBP: Systolic Blood Pressure
SD: Standard Deviation
SPSS: Statistical Package for Social Sciences
TCRI: Total Cardiovascular Risk Index
